# The clinical relevance of healthy neurodevelopmental connectivity in childhood and adolescence: a meta-analysis of resting-state fMRI

**DOI:** 10.3389/fnins.2025.1576932

**Published:** 2025-06-26

**Authors:** Merida Galilea Tapia Medina, Raquel Cosío-Guirado, Maribel Peró-Cebollero, Cristina Cañete-Massé, Erwin Rogelio Villuendas-González, Joan Guàrdia-Olmos

**Affiliations:** ^1^Department of Social Psychology and Quantitative Psychology, University of Barcelona, Barcelona, Spain; ^2^Institute of Complex Systems, University of Barcelona, Barcelona, Spain; ^3^Institute of Neuroscience, University of Barcelona, Barcelona, Spain; ^4^Faculty of Psychology, Universidad Michoacana de San Nicolás de Hidalgo, Morelia, Mexico

**Keywords:** brain connectivity, fMRI, resting-state, meta-analysis, healthy neurodevelopment

## Abstract

**Background:**

In recent years, interest has grown in brain connectivity during infancy and adolescence, particularly in understanding neurodevelopment. Research is focusing on how brain network complexity evolves, providing insight into developmental neural connectivity. While some studies highlight key periods of brain maturation, findings remain inconsistent, leaving the neural correlates of typical development uncertain. This meta-analysis aims to identify brain regions and functional connectivity networks that show age-related activation patterns. Our goal is to clarify how neural wiring and complexity change with age, using seed-based d mapping (SDM) to analyze resting-state functional connectivity.

**Methods:**

We reviewed studies employing resting-state functional magnetic resonance imaging (rs-fMRI) to examine brain connectivity in typically developing children and adolescents. After thoroughly application of the rigorous inclusion criteria, five studies published between 2013 and 2024 remained selected for this analysis. While this is a small number, this limitation reflects our unwavering commitment to methodological rigor and the current scarcity of available literature, ensuring that only high-quality studies were considered.

**Results:**

Consistent increases in seed-based connectivity involving the left frontal and prefrontal cortices were observed, particularly the left superior frontal gyrus and bilateral anterior cingulate cortex. These areas showed increased connectivity in older compared to younger participants.

**Conclusion:**

The left frontal and prefrontal cortices, which are critical for executive function, attention, and intelligence, appear to strengthen their connectivity during childhood and adolescence. These observations provide a preliminary glimpse into typical brain maturation. However, due to the small number of studies and heterogeneity in age comparisons. No clinical implications can be drawn at this stage, and further research is required to confirm these developmental trends.

## 1 Introduction

The human brain comprises numerous functional networks that interact with one another. These systems evolve as significant changes in the functional development of the brain take place between childhood and adolescence (Tian et al., 2023). Neurodevelopmental research on brain maturation seeks to elucidate the organization of these complex neural circuits during development. These investigations can advance our understanding of this window of vulnerability for disruptions of normative development and, consequently, the increased incidence of psychiatric and neuropsychological conditions (Sato et al., 2015; Wainberg et al., 2022). A recent meta-analysis conducted by Solmi et al. (2022) estimated that in almost two thirds of cases, mental disorders emerge before the age of 25. Considering the relevance of such critical stages, the study of these age-related variations in healthy children and adolescents furnishes a fundamental framework for understanding the neurodevelopmental mechanisms of mental disorders.

Measurement of blood oxygen level-dependent signal using functional magnetic resonance imaging (fMRI) is now the gold-standard for tracking neural development in the human brain (Li et al., 2019). Among them, resting-state fMRI (rs-fMRI) is the most commonly used method to study brain developmental trajectories. It reveals the intrinsic functional connectivity (FC) that occurs in the absence of any external input (Razi et al., 2017). This provides exceptionally relevant information about the coupling dynamics and the relationships between networks as it unveils the brain activity driven essentially by spontaneous fluctuations (Buckner et al., 2013). The absence of an external complex task is a benefit of this method: it facilitates data acquisition and requires less participants’ collaboration. Therefore, FC estimates are frequently applied to pediatric population (Bernal, 2022; Buckner et al., 2013). For the reasons exposed, the resting-state paradigm has garnered much attention in the last few years in the field of neurodevelopment.

During adolescence, the functional networks undergo a process of specialization with age, and recent studies indicate that during this stage, the functional connections of the brain restructure. This process of refinement is characterized by increased within-network connectivity and decreased between-network connectivity. This is, functional segregation -or strength in the correlation with near areas- diminishes, while functional integration –or connection with far regions- enhances (Cao et al., 2016; Içer, 2019).

Wierenga et al. (2016) explored developmental patterns during childhood and adolescence and describe fine-tuning of brain organization with less path length and more node strength and network clustering. With age, global and local efficiency in adolescents increases compared to children, which they consider a mediator of the gradual progress of executive functions. Comparing children to adolescents, Tian et al. (2023) concluded that transmission efficiency of the brain gradually incremented as degree centrality and nodal efficiency increased with age and nodal shortest path was reduced. Li et al. (2019) suggest specialization begins as early as 3 to 5 years of age, as during this age period they observe a tendency to strengthen brain connections and to reduce generalization of brain activation.

All in all, it seems that the brain development trajectory in children shares the same core regions as adults. The main differences lie in the number of connections and their strength: less but stronger links appear with age (Içer, 2019; Jolles et al., 2011). The significant age-related shift of FC patterns appears to be associated with frontal regions (Jolles et al., 2011; Kelly et al., 2009). To this date, structural imaging findings have delineated typical brain developmental trajectories; however, the functional changes during this sensitive development period of childhood and adolescence remains less explored. The systematic review carried out by Cosío-Guirado et al. (2024) represents an attempt to understand typical brain development using rs-fMRI. This meta-analysis could help fill in the gap on whether there are discernible developmental patterns using a resting-state fMRI approach and go beyond the results issued by the previous review. Such patterns might help to describe possible preliminary patterns of normative development, which ultimately can contribute both as a baseline and as a comparison point to detect atypical developmental trajectories and diagnose emerging mental and neurological disorders.

We aim to perform a meta-analysis of FC in typically developing children and adolescents studied through resting-state fMRI. The purpose of this meta-analysis is to identify brain regions that show distinctive activation with age at rest. Hence, our goal is to characterize age-related changes and brain maturation in relation to the wiring of neural networks and brain complexity. Based on published literature, we expect to find a pattern of increased FC with age. To this end, we included papers that compared different groups of age and traced their brain connectivity changes, and we identified the combined size effect of the brain connectivity areas, as well as the possible effect of other mediator variables on estimating the effect sizes (sample size, sex, mean age and age range). In this study Seed-based d mapping (SDM) was used, a meta-analysis tool for neuroimaging data that has shown to have high levels of validity and consistency (Albajes-Eizagirre et al., 2021; Shepardson et al., 2023).

## 2 Methods

### 2.1 Literature search and selection criteria

Following PRISMA guidelines (Page et al., 2021), an extensive review was conducted. Articles included in this study were sourced from the PubMed, Web of Science (WoS), and PsycInfo databases, focusing on publications from 2013 to March 2024. The literature search applied a Boolean algorithm combining the following keywords: (“fMRI” OR “functional magnetic resonance imaging”) AND (“children” OR “adolescents” OR “youth” OR “child” OR “teenager”) AND (“resting state” OR “rs-fMRI”) within the title or abstract of the papers.

**Figure 1:**
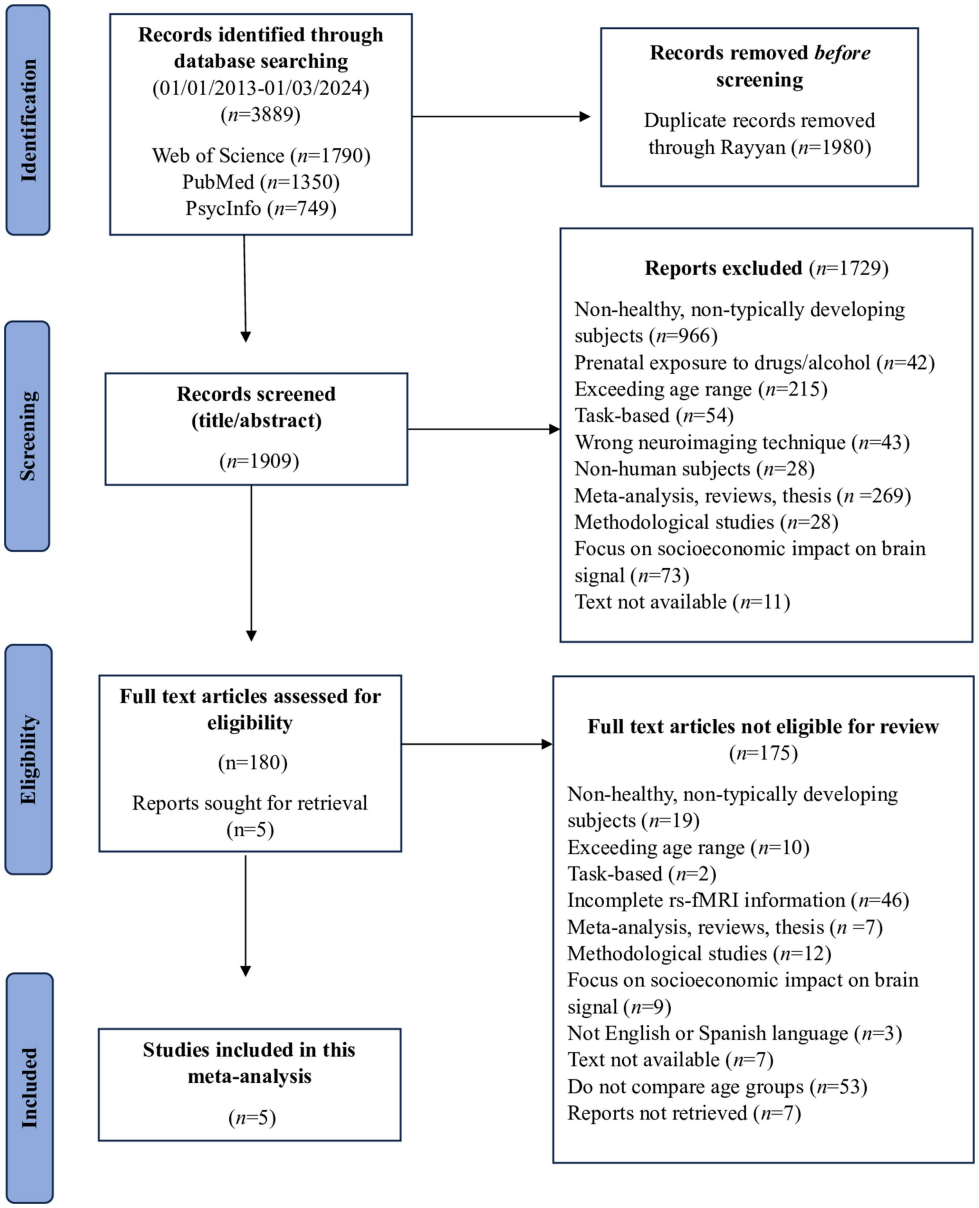
Flowchart of the meta-analysis search conducted.

Two researchers independently conducted the search, resulting in a total of 3,889 studies and reaching 100% agreement. The search yielded 1790 papers from the WoS database, 1,350 from PubMed, and 749 from PsycInfo. Following deduplication across the databases, 1980 duplicate records were removed with the help of Rayyan (Ouzzani et al., 2016). Subsequently, all articles underwent two rounds of screening by two independent reviewers (MGTM, RCG). The initial agreement level during the screening process was 92%, and discrepancies were resolved through discussion. At this stage, 1729 studies were excluded during title/abstract screening due to non-compliance with the inclusion criteria. Following the screening process, 180 articles remained for eligibility assessment. During the eligibility process, 175 studies were excluded due to inconsistencies with the inclusion criteria after the full text had been reviewed. In this phase, agreement reached 93%. Discrepancies were resolved through consensus discussions. As a result, the final sample comprised five papers, which carry the symbol * in the bibliography. Figure 1 provides a visual summary of this search process.

The inclusion criteria were as follows: (a) participants in the study were required to be children and adolescents aged between 3 and 20 years covering early childhood through late adolescence; (b) only healthy or typically developing (TD) individuals were included; (c) the study measured brain signals using fMRI during the resting state and applied a consistent threshold across the entire brain; (d) reported coordinates needed to be in either MNI or Talairach space; (e) only Seed Based approaches were included; and (f) additionally, the studies had to compare two distinct age groups.

We applied the following exclusion criteria: (a) participants had a history or current diagnosis of psychiatric or neurodevelopmental disorders, or if they had serious medical conditions that could affect normal development; (b) studies involving participants with parental histories of major psychiatric or neurological disorders were also excluded, as were those with parent-reported prenatal exposure to alcohol or drugs; (c) task-based approaches and neuroimaging techniques other than fMRI were not considered; (d) systematic reviews, meta-analyses, theses, or clinical essays were excluded from the analysis, as were animal studies; and (e) studies focusing on the impact of socioeconomic conditions, poverty, or parental neglect on brain signals were also excluded.

### 2.2 Data extraction

Relevant information from each article was recorded in a spreadsheet, covering: (1) sample characteristics, detailing the population type (children, adolescent), sample size, gender, and age; (2) resting time during fMRI; (3) established comparisons; (4) study design; (5) signal analysis; (6) examined brain areas; (7) coordinates (MNI or Talairach) and peaks; (8) software used for image preprocessing; and (9) main findings.

### 2.3 Meta-analysis

Voxel-wise meta-analysis was conducted using the Seed-based d Mapping (SDM) software (available at http://www.sdmproject.com) to analyze the differences between two age groups in healthy participants. The approach details are described by Müller et al. (2018) and Radúa and Mataix (2012) with the following parameters: 20 mm FWHM, 2 mm voxel size, 50 imputations, and 1,000 permutations. The reporting thresholds were set at *p* < 0.005 with a cluster size ≥ 10 voxels. First, we selected the reported peak coordinates of all functional differences significant at the whole brain level in these studies. All studies included used the same statistical threshold throughout the whole brain to avoid potential bias towards regions with liberal thresholds. The minimum threshold was defined by a *p*-value of 0.001 and Student’s t reference value, with degrees of freedom (df) estimated by the conventional expression (n1 + n2–2). Second, we recreated peak coordinates for each study with a standard MNI map of the group differences’ effect size based on their peak t value using a non-normalized Gaussian kernel, assigning higher values to the voxels closer to the peaks. Third, we obtained the mean map by voxel-wise calculation of the study maps’ random-effects mean, weighted by the sample size. Fourth, to correctly balance sensitivity and specificity, we used a p-value of 0.05 as the main threshold with an additional peak height of Z = 1.

Lastly, after calculating Cohen’s d and the confidence intervals (CI) for the papers, we conducted a covariate analysis for mean age, total sample size, male percentage, and minimum age. To control for false positives, we used the correction described by Albajes-Eizagirre et al. (2021), based on the permutation of images to minimize error. Additionally, we examined the potential publication or analytic bias, and we found no significant evidence of either of these issues.

### 2.4 Quality assessment

Quality assessment was performed using a 10-point checklist based on previous meta-analyses (Chen et al., 2015; Du et al., 2014; Shepherd et al., 2012; Ruiz-Torras et al., 2023) to evaluate the imaging-specific methodology, demographic and clinical aspects of the included studies. The quality assessment details are shown in Supplementary material. This checklist was not designed as an assessment tool; however, it provided an objective indication of the rigor of the individual studies included. One author reviewed the included articles and determined a rating for each one, another researcher evaluated the resulting scores, and a consensus quality assessment score was obtained. The quality scores for each study are presented in Table 1.

**Table 1:** Demographic characteristics of the participants in data included in this meta-analysis.

Study	*n*		Age studied (mean, standard deviation)			fMRI characteristics	% Males	Analysis	Quality score
	Younger group	Older group	Younger group	Older group	Range				
Dai et al. (2019)	15	15	3 ± 0.16	5 ± 0.16	3–5	8′ (3 T)	50%	Seed-based	9
Içer (2019)	18	17	8.5 ± 0.56	16.9 ± 0.74	8–18	NR (3 T)	45.3%	Seed-based	9
Liuzzi et al. (2023)	34	29	4.4 ± 0.25	6.5 ± 0.30	4–6	7′6″ (3 T)	48.9%	Seed-based	9.5
Xiao et al. (2016a)	15	15	3 ± 0.16	5 ± 0.16	3–5	8′ (3 T)	50%	Seed-based	9.5
Xiao et al. (2016b)	19	17	3 ± 0.16	5 ± 0.16	3–5	8′ (3 T)	47.2%	Seed-based	8.5

NR, not reported; fMRI characteristics include the minutes under resting-state and the number of Teslas of the scanner used; Quality score out of 10.

## 3 Results

### 3.1 Studies included in the meta-analysis

Table 1 presents the data obtained from each study, detailing the mediator variables analyzed in each paper (sample size, percentage of males, mean age, and age range). Four studies focus on the FC of children aged 3 to 6 years, while only one study in this meta-analysis examines children and adolescents aged 8 to 18 years. The total sample size across all studies is 194 participants. Table 1 also provides fMRI characteristics, including resting time, which ranges from 7.6 to 8 min; one study, however, does not report the resting time. Additionally, the table includes information on the magnetic field strength (measured in Teslas) of the MRI machines used in each study. And finally, the score of the quality assessment. All studies in this meta-analysis have addressed FC using seed-based analysis. We have restricted this meta-analysis to a single strategy, seed-based fMRI, to control confounding variables that could introduce “noise” into the results and avoid comparing results obtained through different strategies.

### 3.2 Qualitative study

All the results from each article included in the meta-analysis have been analyzed. Table 2 lists the clusters, maximum and minimum peaks detected by the preprocessing for each study. Additionally, in the Cohen’s d column, the studies with a significant effect size, as well as the associated Z and *p* values, can be found (Albajes-Eizagirre et al., 2021). As it can be observed, out of the five studies included in the meta-analysis sample, only two have obtained a significant effect size. However, the main results of all five studies included in the sample will continue to be presented in order to enrich the results and findings.

**Table 2:** Peak coordinates obtained in the different studies included in the meta-analysis.

Study	Cohen’s *d*	Coordinates MNI	Region	*Z*	*p*	Peak
		x, y, z				
Dai et al. (2019)		−20, −72, 22	Left superior occipital gyrus, BA 18			Min
Içer (2019)		0, −78, −34	Left cerebellum, crus II			Min
Liuzzi et al. (2023)	−1.118	−18, −100, −10	Left inferior occipital gyrus, BA 18	−4.099	<0.001	Min
Xiao et al. (2016a)	−1.442	−46, 12, −18	Left temporal pole, superior temporal gyrus, BA 38	−3.467	<0.001	Max
		−8, −30, 2	Left thalamus			Min
Xiao et al. (2016b)		50, 14, −30	Right temporal pole, middle temporal gyrus, BA 38			Min

The table lists the clusters and peaks detected by the preprocessing for each study. The second column details the studies with a significant effect size. Max, maximum peak; Min, minimum peak.

**Figure 2:**
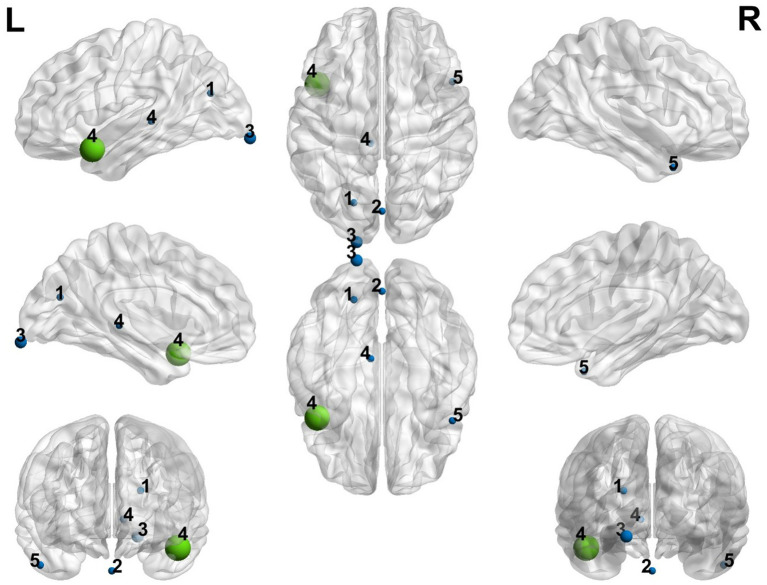
Representation of the positive and negative peak coordinates obtained in the different studies included in the meta-analysis. Blue color represents the minimum peaks and green color represents the maximum peak. The size of every node is proportioned to the Cohen’s d calculated through the meta-analysis. (1) Dai et al. (2019), (2) Içer (2019), (3) Liuzzi et al. (2023), (4) Xiao et al. (2016a), (5) Xiao et al. (2016b).

Figure 2 visualized with the BrainNet Viewer (http://www.nitrc.org/projects/bnv/; Xia et al., 2013) displays the different positive and negative coordinates of the results from each article after preprocessing with SDM. The green node indicates the maximum peak, while the blue nodes represent the minimum peaks. It is also noteworthy that the nodes labelled as 3 and 4 represent studies with a significant effect size. The other effects present non-relevant statistical significance values and very low Cohen’s d values although we included them in the corresponding table and figure for a more exhaustive description.

### 3.3 Meta-analysis results

**Figure 3:**
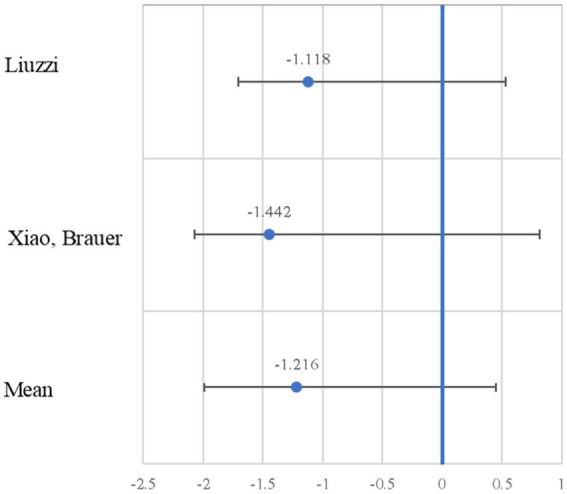
Forest plot of the negative effect size. The effect size (Cohen’s d; blue circle) is shown with the 95% confidence interval.

Figure 3 presents the forest plot of the studies, indicating the effect size and the 95% confidence interval (CI). As can be observed from the sample of five studies, only two show significant effects. The most significant negative effect size is −1.442, with a lower CI of −2.257 and an upper CI of −0.627 (Xiao et al., 2016a). Regularly, in the forest plot of a standard meta-analysis, the effect sizes of both significant and non-significant studies are added. However, in our meta-analysis, the studies cover different brain areas, consequently the meta-analytic process excludes certain information that we believe is relevant to explaining the effect of neurodevelopment. That is why in the following results, we will continue to present the details of the five studies in the sample.

In the meta-analysis, older participants exhibited activation in frontal areas compared to younger participants. Notably, there was decreased activation in the left superior frontal gyrus medial (SFG), specifically in Brodmann area (BA) 9. One cluster comprising 714 voxels was identified. The results of the principal meta-analysis are displayed in Table 3. In the second part of the table can be found the breakdown of the principal cluster.

**Table 3:** Major differences in activation between older participants and younger participants in this meta-analysis.

MNI coordinate	*Z*	*p*	Voxels	Description
−6,46,36	−2.914	0.009	714	Left superior frontal gyrus, medial, BA 9
Local peaks				
−6,46,36	−2.914	0.009	109	Left superior frontal gyrus, medial, BA 9
2,44,38	−2.87	0.009	73	Left superior frontal gyrus, medial
2,46,16	−2.707	0.012	89	Left anterior cingulate / paracingulate gyri, BA 32
6,46,24	−2.598	0.015	87	Right anterior cingulate / paracingulate gyri, BA 32
−4,42,20	−2.574	0.021	89	Left anterior cingulate / paracingulate gyri, BA 32
2,42,26	−2.522	0.015	174	Left superior frontal gyrus, medial, BA 32
−4,56,26	−2.317	0.044	27	Left superior frontal gyrus, medial, BA 10

MNI, Montreal Neurological Institute; BA, Brodman area. The second part of the table corresponds to the breakdown of the cluster.

**Figure 4:**
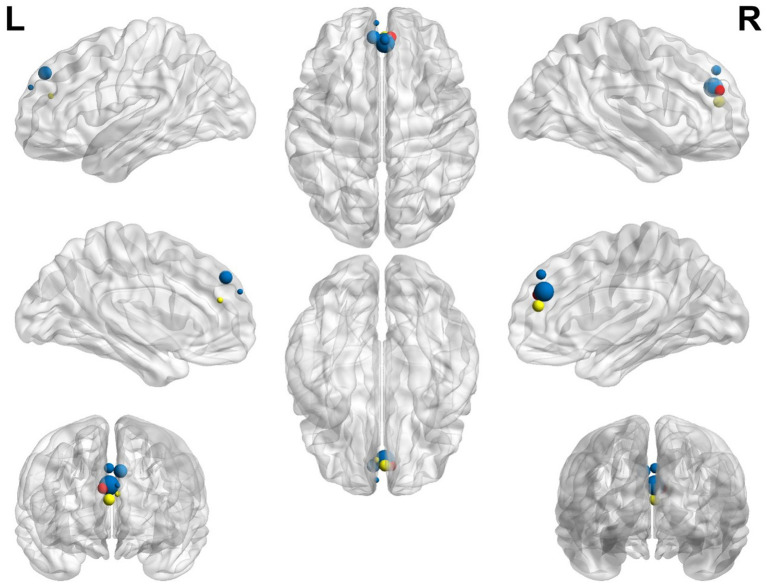
Representation of the most significant coordinates that show activation in older participants compared with younger participants. The left superior frontal gyrus, medial, is shown in blue; the left anterior cingulate/paracingulate gyri is shown in yellow; the right anterior cingulate/paracingulate gyri is shown in red.

The graphical representation of activated areas is shown in Figure 4. The size of each node is proportional to the number of voxels it represents; thus, larger nodes correspond to regions with a greater number of voxels and indicate larger areas. Notably, blue nodes represent the left SFG medial, yellow nodes denote the left anterior cingulate cortex (ACC)/paracingulate gyri (PaCG), and red nodes indicate the right ACC/ PaCG.

When performing the complementary analyses with the covariates –sample size, sex, mean age, and age range- no different results were found from those presented in the main meta-analysis. This allows us to infer that these variables only confirm the importance of frontal areas in the neurodevelopment of healthy children and adolescents. The data from the covariate analysis can be found in Supplementary Table S1.

### 3.4 Heterogeneity analysis

In examining the included studies in this meta-analysis, we found that the value of tau (*τ*) is <0.001, suggesting no heterogeneity in the estimated effect sizes. Additionally, the Q statistic has a value of 0.422, which does not provide significant evidence of heterogeneity. The degrees of freedom (df) are 1, related to the number of studies in the analysis. Finally, the *p*-value is 0.515, indicating no significant evidence of heterogeneity. In summary, based on these data, there appears to be no substantial heterogeneity among the analyzed studies.

## 4 Discussion

The present study integrates previous evidence about brain activity during development in childhood and adolescence measured with fMRI at rest. This article aims to systematize and analyze the current knowledge in this field by pooling rs-fMRI studies that compare different age groups, where age was analyzed as an element associated with connectivity at rest. The main finding is that seed-based connectivity involving the frontal and prefrontal regions was greater in older participants compared with younger ones, specifically in the left SFG medial and bilateral ACC/PaCG.

Frontal and prefrontal circuits are accountable for executive function (EF) and cognitive control (CC), which are often used as synonyms (Friedman and Robbins, 2021). EF is defined as the ability to flexibly select, maintain and act towards goal-oriented information, while suppressing irrelevant stimuli (Silvieira et al., 2021). Impaired EF is known to be a transdiagnostic feature of several mental disorders (McTeague et al., 2016) and it has been proven to predict psychopathology in children (Romer and Pizzagalli, 2021). Although EF begin to develop during childhood, its improvement and refinement continue through adolescence and even early adulthood (Luna et al., 2015). Hence, this period plays a crucial role in determining the trajectory toward either healthy or impaired neurocognitive development.

Research over the past decade has revealed ACC as a major neural hub for EF; specifically, attention control, error detection, reward and error prediction, response selection, and conflict monitoring (Vassena et al., 2017; Vassena et al., 2020). Also, Gennari et al. (2018) suggest that ACC/PaCG activity modulates attention and stimulus encoding in order to optimize performance. Evidence underlines the role of ACC in decision-making and flexible behavioral patterns: it drives pursuing more rewarding options, even when more effort is required, along with updating predictive models in changing contexts (Brown and Alexander, 2017; Monosov and Rushworth, 2022; Vassena et al., 2020). Altogether, this alludes to a more refined process of decision-making where ACC leads to effortful behavior as well as disengagement from less adaptative behaviors (Brown and Alexander, 2017). This might respond to the major need of humans to adapt their behavior to a rapidly changing environment requiring speed and flexibility to evaluate stimulus and feedback, in order to give an adequate behavioral response. These environmental demands greatly escalate during the early years, which may explain the peak activity of ACC observed in this meta-analysis. In addition, preparing for a cognitively effort-demanding task is linked to increased ACC activity, as described by Vassena et al. (2014). Our results seem consistent with this idea, since we observe higher activation within the ACC in the older youth compared to the younger ones and, presumably, more cognitively demanding tasks are expected to appear with age.

SFG has received limited research attention. This region has shown to be associated with cognitive functions such as working memory (Maconeavu et al., 2023; Rottschy et al., 2012), executive cognitive control (Maconeavu et al., 2023), and problem-solving and decision-making (Barretto-García et al., 2024). SFG activation has also shown to be implied in better ability to suppress intrusive thoughts, a trait of certain psychiatric disorders, including neuroticism, depression and attention-deficit/hyperactivity disorder (Lu et al., 2022).

A very relevant goal of research within the neurodevelopmental field is to identify the sensitive brain areas that indicate healthy and unhealthy development. Some emerging studies point to ACC and EF as one of the brain regions and cognitive functions, respectively, that influence the typical healthy development of children and adolescents. Silvieira et al. (2021) conclude that FC of the dorsal ACC (dACC) mediates the development of externalizing and internalizing psychopathology, in a group of 12–22 years old adolescents. They found that childhood adversity was negatively associated with activation of the dACC. Similarly, Maggioni et al. (2024) studied a sample of children and adolescents with emotional-behavioral problems, and they noticed a link between prenatal complications, grey matter volume reductions in the right SFG and ACC, and withdrawn problems.

Taken together, there is accumulated evidence that supports that the importance of frontal areas in development, specifically the SFG and ACC might be considered as key regions within the delicate developmental phase. However, given the limited and heterogeneous age range no general conclusions can be drawn about executive function, frontal activation or clinical implications from this work. Moreover, most studies to this date are based on already impaired children and use task-based fMRI. More studies are needed in the healthy population of children and adolescents from the paradigm of rs-fMRI.

The papers included were considered high-quality and we believe it is appropriate to promote the use of rs-fMRI to explain cognitive neurodevelopment and brain maturation. We are aware that the sample for this meta-analysis is very limited and this is due to two main reasons. One, the little evidence available in science nowadays regarding typical development studied with fMRI at rest. Traditionally, this approach of studying healthy maturation has been overshadowed by the research of the pathological brain. Hence, the current evidence in this matter is scarce. Two, with aims of preserving the methodological quality and offering reliable results, we established strict inclusion and exclusion criteria. This decision necessarily reduced the final sample for this work. Therefore, we acknowledge that it is currently optimistic to claim generalized conclusions, in view of the little evidence available. However, we believe that the conclusions derived from this meta-analysis reflect the reality of the current state of the art.

After the extensive literature review we have performed for this meta-analysis, we aim to provide useful indications to support future works based on rs-fMRI with children and adolescents.

First, it is acknowledged that reducing noise and head movement effects is a prominent concern in the field of fMRI, especially when working with pediatric populations, where there is an inverse relationship between head motion and age (Fassbender et al., 2017). Large head motion can lead to severe noise that results in useless recordings or inflates correlation between adjacent brain areas and disregards correlations between distant regions. In this regard, the recommendations by Bernal (2022), Ciric et al. (2017), and Greene et al. (2016) are a great guide for reducing the undesirable effects of high-movement populations such as children. To avoid these movement effects, training the children for staying in the resonator should be the first option. Children and adolescents can be anxious during unfamiliar and uncomfortable situations such as an fMRI scanner. It has been demonstrated that a training session in a fMRI mock scanner effectively suppresses head motion (Gao et al., 2023), improves patient compliance and reduces anxiety (Durston et al., 2009). Furthermore, a potentially useful approach that can be added to the mock scanner is promoting fantasy surrounding the task. Only one study in this meta-analysis, by Liuzzi et al. (2023) trained their children for the scanner. Once the recordings are obtained, there are several alternatives to correct head movement effects. One, discarding participants whose mean displacement values are above the 1.5-mm threshold of framewise displacement for 3-mm isotropic voxels, as suggested by Soares et al. (2016). Two, applying certain statistical adjustments. Scrubbing regression has emerged as an effective choice at removing spurious sources of activity in fMRI data. This method removes those repetition times exceeding 0.2 Jenkinson’s framewise displacement, according to Yan et al. (2013) and Power et al. (2012, 2013). Three, analyzing head movement as a nuisance covariate, as used by Cañete-Massé et al. (2023) in a delicate population like Down Syndrome. The innovative motion correction method developed by Steinbrenner et al. (2023) for pediatric epilepsy patients should also be considered for all pediatric population. All five studies included apply some type of motion artifact statistical correction, mainly framewise displacement, and covariation with head movement parameters from realignment.

A second recommendation is to include total brain volume as a covariate in rs-fMRI developmental studies. As it is known, brain structures grow in volume with age (Groeschel et al., 2010; Mills et al., 2016). This introduces a potential confounding factor of whether the increase in connectivity with age is due to the increase in volume. Therefore, we reiterate the control of total brain volume as a covariable in rs-fMRI developmental studies because it can have important perverse effects over brain measurements. Power et al. (2012) already recommended the inclusion of gray/white brain matter as a covariable to use in regression analysis of nuisance variables. Recently, some rs-fMRI studies have implemented it, such as and Chen et al. (2022) in young adults and Montalà-Flaquer et al. (2023) in healthy ageing. None of the papers in this meta-analysis register total brain volume and correlate it with the significant brain areas observed in their data.

A third point might be the lack of clarity when explaining the fMRI analysis strategy employed. The analysis of the rs-fMRI signal can be performed from several approaches, including seed-based, independent component analysis (ICA), amplitude of low-frequency fluctuations (ALFF) and fractional ALFF (fALFF), and graph theory. We observe that several papers do not explicitly mention the strategy they use, and it is for the reader to infer the one they are following, which affects the replicability of the investigation. In this work, we only included seed-based fMRI, as it is not generally recommended to mix results pooled from different analysis strategies.

Fourth, there is a lack of consensus on how the resting-state paradigm in fMRI should be applied. The different interpretations lead to some studies to use rs-fMRI while children are naturally asleep in the resonator (Dai et al., 2019; Xiao et al., 2016a,b), or awake (Içer, 2019), or movie-watching (Liuzzi et al., 2023), or even sedated. The challenge of using rs-fMRI with children and adolescents implies that applying the classic resting-state paradigm is sometimes troublesome–awake, with eyes opened and fixed on a cross symbol on the screen but refraining from thinking anything particular-. For this reason, other applications of rs-fMRI have arisen. Some authors argue that within-scanner sleep is a source of variance in network connectivity and suggest careful monitoring and correction if wakefulness is not possible (Soehner et al., 2019). Movie-watching inside the scanner reduces movement in high movers, however, the selection of the film must follow careful considerations not to cause uncontrolled noise. Vanderwal et al. (2015) propose a movie paradigm that was designed to provide enough stimulation to improve compliance related to motion and wakefulness while reducing cognitive load during data collection. Their movie proposal cause FC patterns that closely resemble awake rest, compared with conventional movies. As an exceptional resource, sedation might be required with children. Sedated rs-fMRI images should be interpreted with greater caution as it produces alterations: it globally disconnects low-level functional networks, while simultaneously increases within connectivity (Hassanzadeh et al., 2023; Wein et al., 2024). This lack of consensus challenge researchers to decide what approach of rs-fMRI they consider more suitable considering their study goals and their sample idiosyncrasy.

As a fifth point, we underline that the sample sizes are small. This is a recurring issue in studies working with children, but the sampling obstacles intensify in the neuroimaging field, thus reducing the reliability of findings. Task-based neuroimaging approaches entail more difficulties in children than resting-state paradigms; however, the small sample problem is evident in this work –sample sizes vary from 30 to 63 participants-.

Along the line of the last point, most developmental studies nowadays cover small age ranges, and hence, few developmental stages. This hinders obtaining useful results since they compare groups of age that are very close to one another. Except for Içer (2019), who covers 8–18 years old, the rest of the papers included work with very small periods of age: 3–5 years of age (Dai et al., 2019; Xiao et al., 2016a,b) and 4–6 years of age (Liuzzi et al., 2023). We advocate for studying broader periods of age, covering various stages of development, from early childhood to late adolescence.

Another challenge is the lack of consensus on the specific age ranges that define each developmental stage. This absence of agreement induces to mix adolescents and children in the same group, and therefore, this distorts the comparisons that we make, and the results obtained from them. There is a need for more clear segmentation of developmental stages and age groups, so that these do not depend on the researcher subjectivity or availability of participants.

As a final point, it is surprising that longitudinal rs-fMRI studies in youth are scarce. Longitudinal studies provide exceptionally important information as they track the changes that neural networks experience throughout the normal development. In this meta-analysis, only Liuzzi et al. (2023) follows a longitudinal perspective and it covered a short age range, yet. These short-ranged longitudinal studies do not exploit the full potential that the nature of these investigations has to unveil the expected neurodevelopment of youth in terms of brain complexity. From of our perspective, performing a cross-sectional evaluation instead of following the changes as in a longitudinal study seems almost a contradiction when the main goal of a developmental study is, precisely, to assess development. The work of Telzer et al. (2018) is a valuable resource for methodological considerations on developmental longitudinal fMRI research.

There are some limitations to this meta-analysis. The first key limitation is that, of the 3,889 studies that were initially identified, only five remained eligible for inclusion. This reflects the scarcity of studies tracking typical development with fMRI at rest. Second, and due to the limited data, none of the variables we considered as potential mediators showed a significant effect (sample size, sex, mean age and age range). Third, we acknowledge that only two of five included studies showed statistically significant effects. As such, these findings must be interpreted with caution and considered preliminary. Nonetheless, it is important to emphasize that one of the main strengths of meta-analytic approaches is precisely their capacity to identify consistent patterns across studies, even when individual studies do not reach statistical significance. Another point to take into account is the great age variability. The inclusion of studies with differing age splits, particularly the broader range in Içer (2019), introduces variability that may obscure non-linear patterns of brain maturation, limit direct comparisons and may reduce precision of developmental inferences. While our results suggest general age-related changes, we acknowledge that these may not reflect a linear progression and caution against overinterpreting them as such. Future longitudinal or multi-point cross-sectional studies are warranted to explore non-linear developmental trajectories. Fifth, we did not include studies that did not report the three-dimensional activation brain coordinates and/or studies that did not report the corresponding statistical contrasts associated with those coordinates. Thus, it is worth mentioning that the available studies allow us to draw some conclusions that need to be taken with caution. The validity of the findings should be further tested in larger samples.

Our work also has some strengths. To the best of our knowledge, this is the first meta-analytic study that approaches healthy neurodevelopment activation using a rs-fMRI approach. We must note that two independent researchers carried out a thorough search of the literature following the PRISMA guidelines. Finally, the fact that the final number of studies included is small responds to an accurate and rigorous process of selection of studies. Finally, the quality of the studies was assessed, and they were all judged high quality.

## 5 Conclusion

In summary, our results showed that seed-based connectivity involving the frontal lobe, in particular the left SFG medial and bilateral ACC/PaCG, was greater in older compared with younger participants. Ultimately, we suggest that measures of brain activation during resting-state may represent an index of brain maturation that allow us to track the underlying processes of healthy typical neurodevelopment. However, these observations are based on a small and heterogeneous sample. Therefore, while our study provides early insight into normative brain development in healthy children and adolescents, it does not support of clinical applications or generalizable conclusions at this stage. From a methodological perspective, we have identified key challenges in studying resting-state FC in pediatric populations and offered some notable points to guide future research in this field. We acknowledge that the number of studies included in this meta-analysis is relatively small, reflecting the scarcity of literature on heathy development using rs-fMRI. Despite these limitations, we believe the results in this meta-analysis provides valuable foundation for future research in this area, but we caution against extrapolating beyond the data presented. Methodologically this study serves as a foundation for future research indicating the importance of including larger sample sizes, more diverse participant groups, and longitudinal designs. Moreover, conducting multimodal meta-analyses or subgroup comparisons based on different fMRI strategies may prove beneficial. Therefore, we argue that the conclusions drawn from this meta-analysis hold certain substantial scientific relevance and hope to contribute to the ongoing discourse in the field. Although our results may offer a glimpse into typical brain maturation and they could potentially inform efforts to differentiate between typical and atypical developmental trajectories, further research, particularly involving clinical populations and symptom data, is needed to better understand their potential clinical relevance and how they might contribute to diagnostic practices or treatment strategies for neurodevelopmental disorders.

## Data Availability

The original contributions presented in the study are included in the article/Supplementary material, further inquiries can be directed to the corresponding author.
