# Supplementary material for: The clinical relevance of healthy neurodevelopmental connectivity in childhood and adolescence: a meta-analysis of resting-state fMRI
Source: Front Neurosci. 2025 Jun 26;19:1576932. doi: 10.3389/fnins.2025.1576932 (PMC12241014; doi:10.3389/fnins.2025.1576932)
Supplement: Supplementary file 1 [file Table_1.DOCX]

Supplementary Material

# Supplementary Appendix S1

**Quality assessment checklist (score 0/0.5/1 per item total score out of 10)**

Category 1: Participants

1. Healthy participants were evaluated prospectively, psychiatric and medical illnesses were excluded.
2. Important variables (e.g., age, sex, illness duration, onset, medication status, comorbidity, severity of illness) were checked either by stratification or statistically.
3. Sample size per group > 10.

Category 2: Methods for image acquisition and analysis

1. Magnet strength ≥ 1.5 Tesla.
2. MRI slice-thickness ≤ 3 mm and more than 1 slice was identified and traced.
3. Coordinates reported in a standard space.
4. The imaging technique used was clearly described so that it could be reproduced.
5. Measurements were clearly described so that they could be reproduced.

Category 3: Results and conclusions

1. Statistical parameters for significant and important non-significant differences were provided.
2. Conclusions were consistent with the results obtained and the limitations were discussed.

# Table S1

*Major differences in activation between older participants and younger participants in this meta-analysis with covariates.*

| **MNI coordinate** | **SDM-Z** | **P** | **Voxels** | **Description** |
| --- | --- | --- | --- | --- |
| Mean age of younger group | | | | |
| -4,38,28 | -3.17 | 0.005 | 634 | Left superior frontal gyrus, medial, BA 32 |
| Mean age of older group | | | | |
| 46,-44,-10 | 0.997 | ~0 | 52456 | Right inferior network, inferior longitudinal fasciculus |
| 38,-36,36 | 0.171 | ~0 | 102 | Right superior longitudinal fasciculus II |
| -66,-10,12 | 0.13 | ~0 | 47 | (undefined), BA 22 |
| 62,-56,36 | 0.179 | ~0 | 21 | Right angular gyrus |
| 16,24,-2 | 0.162 | ~0 | 12 | Right caudate nucleus |
| -16,-6,26 | 0.197 | ~0 | 17 | Corpus callosum |
| 18,30,8 | 0.105 | ~0 | 16 | Corpus callosum |
| 10,-22,28 | 0.082 | ~0 | 12 | Corpus callosum |
| -46,-38,28 | 0.045 | ~0 | 14 | Left superior longitudinal fasciculus III |
| 26,-58,-28 | 0.045 | ~0 | 18 | Right cerebellum, hemispheric lobule VI, BA 37 |
| -12,14,42 | 0.045 | ~0 | 10 | Corpus callosum |
| Total Sample | | | | |
| -6,46,28 | -3.793 | 0.00099999 | 818 | Left superior frontal gyrus, medial, BA 32 |
| Sex (Male percentage) | | | | |
| 0,46,30 | -5.182 | 0.00099999 | 786 | Left superior frontal gyrus, medial, BA 9 |
| Minimun Age | | | | |
| 2,38,26 | -3.335 | 0.00199997 | 862 | Left anterior cingulate / paracingulate gyri, BA 32 |
